# Implication of cognitive-behavioral stress management on anxiety, depression, and quality of life in acute myocardial infarction patients after percutaneous coronary intervention: a multicenter, randomized, controlled study

**DOI:** 10.1007/s11845-023-03422-6

**Published:** 2023-06-23

**Authors:** Biqun Chen, Juanling Wen, Deyi You, Yu Zhang

**Affiliations:** 1 Intensive Care Unit, Xiamen University, Zhongshan Hospital, Xiamen, 361004 China; 2 Otolaryngology Head and Neck Surgery, Xiamen University, Zhongshan Hospital, Xiamen, 361004 China; 3 Department of Nursing, Xiamen Cardiovascular Hospital Xiamen University, No. 2999 Jinshan Road, Xiamen, 361006 China

**Keywords:** Acute myocardial infarction, Anxiety and depression, Cognitive-behavioral stress management, Percutaneous coronary intervention, Quality of life

## Abstract

**Objective:**

Cognitive-behavioral stress management (CBSM) intervention enhances the psychological status and quality of life in patients with various diseases, such as cancer, human immunodeficiency virus infection, chronic fatigue syndrome, and multiple sclerosis. This multicenter, randomized, controlled study intended to explore the potential benefit of CBSM in ameliorating the anxiety, depression, and quality of life (QoL) in acute myocardial infarction (AMI) patients after percutaneous coronary intervention (PCI).

**Methods:**

A total of 250 AMI patients who received PCI were randomly allocated to the CBSM (*N* = 125) and control care (CC) (*N* = 125) groups, and underwent weekly corresponding interventions for 12 weeks. The hospital anxiety and depression scale (HADS), EuroQol 5D (EQ-5D), and EuroQol visual analogue scale (EQ-VAS) scores were evaluated at baseline (M0), month (M)1, M3, and M6. Major adverse cardiovascular events (MACE) were recorded during follow-up.

**Results:**

HADS-anxiety score at M1 (*P* = 0.036), M3 (*P* = 0.002), and M6 (*P* = 0.001), as well as anxiety rate at M6 (*P* = 0.026), was reduced in the CBSM group versus the CC group. HADS-depression score at M3 (*P* = 0.027) and M6 (*P* = 0.002), as well as depression rate at M6 (*P* = 0.013), was decreased in the CBSM group versus the CC group. EQ-5D score at M3 (*P* = 0.046) and M6 (*P* = 0.001) was reduced, while EQ-VAS score at M1 (*P* = 0.037), M3 (*P* = 0.010), and M6 (*P* = 0.003) was raised, in the CBSM group versus the CC group. However, accumulating MACE rate did not differ between the two groups (*P* = 0.360).

**Conclusion:**

CBSM ameliorates anxiety, depression, and QoL but does not affect MACE in AMI patients after PCI.

**Supplementary Information:**

The online version contains supplementary material available at 10.1007/s11845-023-03422-6.

## Introduction

Acute myocardial infarction (AMI) is a crucial cause of morbidity and mortality worldwide, which is divided into ST-segment elevation myocardial infarction (STEMI) and non-STEMI (NSTEMI) [1]. The global incidence of AMI varies across different regions and is influenced by various factors such as age, gender, and lifestyle [2]. Percutaneous coronary intervention (PCI) is an efficacious treatment option for AMI, and its application has improved the prognosis of AMI patients to a certain extent [3]. Unfortunately, disease recurrence and other major adverse cardiovascular events (MACE) would also happen in AMI patients even after successful PCI [4–6]. It is estimated that the incidence of recurrent AMI after PCI within 3 years ranges from 3.6 to 6.9% [4, 7], and the incidence of MACE after PCI within 2 years ranges from 9.7 to 16.5% [8–10]. The worry of AMI, as well as the fear of disease recurrence and MACE, places a tremendous psychological burden (such as anxiety and depression) and affects the quality of life in AMI patients who undergo PCI, which may further influence their clinical outcomes [11–14]. As a result, exploring potential interventions to enhance the psychological status and quality of life in AMI patients who undergo PCI is necessary.

Cognitive-behavioral stress management (CBSM) is a type of psychotherapeutic intervention, which helps people learn how to deal with destructive thoughts or negative emotions [15, 16]. In the recent decade, CBSM has been disclosed to have a certain benefit to ameliorate mental health and the quality of life in patients with various diseases, such as cancers, human immunodeficiency virus infection, and chronic fatigue syndrome [16–19]. Unfortunately, evidence regarding the benefit of CBSM intervention on attenuating anxiety and depression, along with increasing the quality of life in heart disease patients, is scarce. The only existing study reports that CBSM intervention not only reduces fear of recurrence and stress levels but also improves well-being in atrial fibrillation patients [20]. However, the potential benefit of CBSM intervention in AMI patients who receive PCI has not been studied yet and deserves investigation.

Accordingly, the current research aimed to investigate the implication of CBSM intervention in relieving anxiety and depression, along with enhancing the quality of life in AMI patients who received PCI.

## Methods

### Patients

In this randomized, controlled study, two hundred and fifty AMI patients who received PCI treatment between February 2020 and June 2022 were enrolled, and the treatment of PCI for AMI patients is following a guideline [21]. The inclusion criteria were as follows: (1) diagnosis with AMI according to the 3^rd^ universal definition of myocardial infarction (MI) [22], (2) aged > 18 years, (3) had the ability to complete assessments, (4) willing to cooperate with the completion of the questionnaire related to this study. The exclusion criteria were as follows: (1) with malignant diseases, (2) with a severe mental disorder or cognitive impairment that could not communicate normally. The Ethics Committee approved this study. Informed consent was collected from each patient.

### Data collection and randomization

Clinical features of AMI patients were collected, which included demographics, history of chronic diseases, disease-related information, laboratory tests, and PCI-related information. After enrollment, all patients were randomly assigned into the CBSM or control care (CC) groups using a 1:1 ratio. The randomization was conducted by the block randomization method (bloke size = 4). Random grouping information for each patient was sealed in an opaque envelope, and which patient’s ID was written on the cover. In chronological order of patient enrollment, the patient was given the opaque envelope and allocated to two different groups.

### Care intervention

After randomization, the interventions were carried out by the trained nurses on both CC and CBSM groups for 12 weeks in a team form (8–10 patients per team). Patients assigned to teams based on the order of discharge time and group information. Each intervention was lasted 120 min, and was conducted on the morning of the first Saturday of each week by trained nurses. Notably, the day before each intervention, trained nurses at their respective centers would inform the time and place of the interventions by sending short message service (SMS) texts and emails to remind patients. If patients were initially unresponsive, the trained nurses would subsequently remind them by telephone. In addition, there was no uniform location for the intervention; the patients participated in the intervention at their corresponding centers.

For the CC group, the intervention contained a 60-min presentation centered on education related to basic knowledge of AMI, postoperative care, rehabilitation, diet, and physical exercise. Following that, a 30-min question-and-answer session and another 30-min free time were conducted.

For the CBSM group, after a 60-min presentation with the same content as the CC group, a specific intervention including a 30-min CBSM skills-teach session and another 30-min CBSM-based relaxation training session was followed. In brief, the 30-min CBSM skills-teach session mainly included the following aspects: (1) stress identification and cognitive reconstruction, which was developed by guiding and encouraging patients to talk about their current problems and stresses; (2) emotion management and confidence building, which was developed by guiding patients to express their emotional changes and the reasons. Besides, the 30-min CBSM-based relaxation training session included deep breathing, meditation, and muscle relaxation.

### Evaluation

The hospital anxiety and depression scale (HADS) score, EuroQol 5D (EQ-5D) score, and EuroQol visual analogue scale (EQ-VAS) score were evaluated at baseline (M0), 1^st^ month (M1), 3^rd^ month (M3), and 6^th^ month (M6). The HADS score was used to evaluate patients’ anxiety and depression, which was scaled from 0 to 21 for each aspect (the higher the worse) [23]. The HADS was a Chinese version, and intraclass correlation coefficient (ICC) for total scale, HADA-anxiety (HADS-A), and HADS-depression (HADS-D) was 0.945, 0.921, and 0.932, respectively [24]. The EQ-5D and EQ-VAS scores were applied to evaluate the quality of life, in which EQ-5D score ranged from 5 to 15 (the higher the worse) and EQ-VAS score ranged from 0 to 100 (the higher the better) [25]. The EQ-5D and EQ-VAS was also a Chinese version, and the ICC for EQ-5D and EQ-VAS was 0.79 and 0.80, respectively [26]. Besides, patients also underwent routine follow-ups for 6 months, and MACE was recorded, which was defined as cardiovascular death, myocardial infarction, unplanned coronary revascularization, and hospital admission for cardiovascular cause [27]. The primary outcome in this study was the HADS-A score assessed at M6. The secondary outcomes included HADS-D score at M6, EQ-5D score at M6, EQ-VAS score at M6, and MACE.

### Statistics

According to clinical experience, the sample size calculation was performed per the hypothesis that the mean HADS-A at M6 in the CBSM group was 6, while the mean HADS-A at M6 in the CC group was 7. The standard deviation (SD) was supposed as 2.3. With the significance (*α*) level of 0.05 and the power of 85%, the minimum sample size was 96 for each group and then adjusted to 125 considering the drop-out possibility of 20%. Comparisons between the two groups were assessed by the student *t*-test and *χ*^2^ test. The Kaplan–Meier curve was used to show accumulating MACE rate, and log-rank test was utilized for comparing the difference between two groups. *P* < 0.05 indicated significance. SPSS v.26.0 (IBM, USA) was used for data processing and GraphPad Prism v.7.0 (GraphPad Software, USA) was used for figure plotting.

## Results

### Study flow

**Fig. 1:**
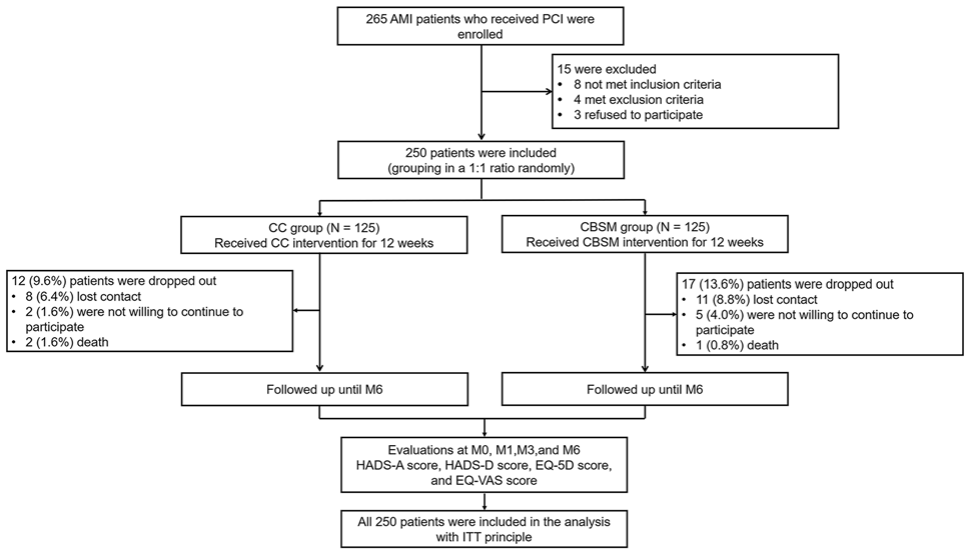
Study process

Initially, 265 AMI patients who received PCI were screened, and 15 patients were excluded, containing 8 patients who failed to meet the inclusion criteria, 4 patients who met the exclusion criteria, and 3 patients who refused to participate. Then, 250 patients were included and randomly allocated to CC and CBSM groups in a 1:1 ratio. In the CC group (*N* = 125), patients received CC intervention for 12 weeks, and 12 (9.6%) patients dropped out, including 8 (6.4%) patients who lost contact, 2 (1.6%) patients who were not willing to continue to participate in this study, and 2 (1.6%) patients who died. In the CBSM group (*N* = 125), patients received CBSM intervention for 12 weeks as well, and 17 (13.6%) patients dropped out, including 11 (8.8%) patients who lost contact, 5 (4.0%) patients who were not willing to continue to participate in this study, and 1 (0.8%) patient who died. Patients in both groups were followed up until M6. The HADS-A score, HADS-D score, EQ-5D score, and EQ-VAS score were assessed at M0, M1, M3, and M6. All patients were included in the analysis with the intention-to-treat (ITT) principle (Fig. 1).

### Clinical characteristics of CBSM and CC groups

The mean ages of the CBSM group and the CC group were 62.8 ± 10.2 years and 63.6 ± 9.7 years, respectively (*P* = 0.525). Meanwhile, there were 38 (30.4%) females and 87 (69.6%) males in the CBSM group, as well as 30 (24.0%) females and 95 (76.0%) males in the CC group (*P* = 0.256). Other clinical features were not different between the two groups either (all *P* > 0.05). Notably, HADS-A, HADS-D, EQ-5D, and EQ-VAS scores at baseline were also not different between the two groups (all *P* > 0.05). Specific clinical information of AMI patients who received PCI is listed in Table 1.

**Table 1:** Clinical features of AMI patients

Features	CC group (*N* = 125)	CBSM group (*N* = 125)	*P* value
Age (years), mean ± SD	63.6 ± 9.7	62.8 ± 10.2	0.525
Gender, No. (%)			0.256
Female	30 (24.0)	38 (30.4)	
Male	95 (76.0)	87 (69.6)	
BMI (kg/m^2^), mean ± SD	25.4 ± 3.3	25.0 ± 3.4	0.289
Marital status, No. (%)			0.471
Married	95 (76.0)	90 (72.0)	
Single/divorced/widowed	30 (24.0)	35 (28.0)	
Employment status, No. (%)			0.884
Employed	32 (25.6)	31 (24.8)	
Unemployed	93 (74.4)	94 (75.2)	
Education level, No. (%)			0.489
Primary school or below	30 (24.0)	24 (19.2)	
Middle or high school	60 (48.0)	69 (55.2)	
Undergraduate or above	35 (28.0)	32 (25.6)	
Location, No. (%)			0.539
Urban	96 (76.8)	100 (80.0)	
Rural	29 (23.2)	25 (20.0)	
Smoker, No. (%)			0.601
No	76 (60.8)	80 (64.0)	
Yes	49 (39.2)	45 (36.0)	
History of hypertension, No. (%)			0.893
No	41 (32.8)	42 (33.6)	
Yes	84 (67.2)	83 (66.4)	
History of hyperlipidemia, No. (%)			0.073
No	65 (52.0)	79 (63.2)	
Yes	60 (48.0)	46 (36.8)	
History of DM, No. (%)			0.764
No	95 (76.0)	97 (77.6)	
Yes	30 (24.0)	28 (22.4)	
Clinical manifestation, No. (%)			0.445
NSTEMI	30 (24.0)	25 (20.0)	
STEMI	95 (76.0)	100 (80.0)	
Symptom-to-balloon time (h), median (IQR)	3.8 (2.3–7.0)	3.7 (2.7–6.6)	0.573
WBC (10^9^/L), median (IQR)	9.8 (7.7–12.9)	10.1 (7.9–12.7)	0.879
FBG (mmol/L), median (IQR)	5.5 (4.5–6.3)	5.2 (4.4–6.5)	0.396
Scr (μmol/L), median (IQR)	85.2 (71.0–102.4)	83.1 (72.5–99.9)	0.602
TG (mmol/L), median (IQR)	1.6 (1.0–2.4)	1.8 (1.0–2.4)	0.838
TC (mmol/L), median (IQR)	4.6 (3.8–5.5)	4.7 (3.9–5.3)	0.598
LDL-C (mmol/L), median (IQR)	3.1 (2.3–4.0)	3.1 (2.3–3.9)	0.751
HDL-C (mmol/L), median (IQR)	1.0 (0.9–1.2)	1.1 (0.9–1.3)	0.125
CRP (mg/L), median (IQR)	4.4 (3.2–6.3)	5.1 (3.2–6.3)	0.318
cTnI (ng/mL), median (IQR)	4.6 (3.2–6.4)	4.4 (3.0–6.4)	0.557
CK-MB (ng/mL), median (IQR)	29.5 (17.4–49.9)	32.9 (22.5–52.0)	0.064
Culprit lesion, No. (%)			0.108
LDA	50 (40.0)	61 (48.8)	
LCX	32 (25.6)	19 (15.2)	
RCA	43 (34.4)	45 (36.0)	
Multivessel disease, No. (%)			0.373
No	66 (52.8)	73 (58.4)	
Yes	59 (47.2)	52 (41.6)	
Thrombus aspiration, No. (%)			0.169
No	93 (74.4)	102 (81.6)	
Yes	32 (25.6)	23 (18.4)	
Number of implanted stents, No. (%)			0.767
1	94 (75.2)	96 (76.8)	
2	31 (24.8)	29 (23.2)	
Type of stent, No. (%)			0.579
Sirolimus-eluting stent	90 (72.0)	86 (68.8)	
Everolimus-eluting stent	35 (28.0)	39 (31.2)	
Stent diameter (mm), median (IQR)	3.0 (3.0–3.5)	3.0 (3.0–3.5)	0.219
Stent length (mm) (total), median (IQR)	33.0 (23.0–38.0)	33.0 (23.0–38.0)	0.843
Infarct size (%), median (IQR)	22.0 (17.0–28.0)	24.0 (17.0–30.0)	0.532
HADS-A score, mean ± SD	7.8 ± 2.5	8.0 ± 2.8	0.601
HADS-D score, mean ± SD	7.7 ± 2.6	7.8 ± 2.7	0.685
EQ-5D score, mean ± SD	11.1 ± 1.5	11.3 ± 1.6	0.522
EQ-VAS sore, mean ± SD	61.4 ± 15.1	60.2 ± 16.1	0.571

Comparisons of clinical features between the two groups were assessed by the student *t*-test, *χ*^2^ test, and Wilcoxon test

*AMI* acute myocardial infarction, *CC* control care, *CBSM* cognitive-behavioral stress management, *SD* standard deviation, *BMI* body mass index, *DM* diabetes mellitus, *IQR* interquartile range, *NSTEMI* non-ST-elevation myocardial infarction, *STEMI* ST-elevation myocardial infarction, *WBC* white blood cell, *FBG* fasting plasma glucose, *Scr* serum creatinine, *TG* triglyceride, *TC* total cholesterol, *LDL-C* low-density lipoprotein cholesterol, *HDL-C* high-density lipoprotein cholesterol, *CRP* C-reactive protein, *cTnI* cardiac troponin I, *CK-MB* creatine kinase MB, *LDA* left anterior descending branch, *LCX* left circumflex artery, *RCA* right coronary artery, *HADS-A* the hospital anxiety and depression scale for anxiety, *HADS-D* the hospital anxiety and depression scale for depression, *EQ-5D* EuroQol 5D, *EQ-VAS* EuroQol visual analogue scale

### Comparison of anxiety and depression between CBSM and CC groups

**Fig. 2:**
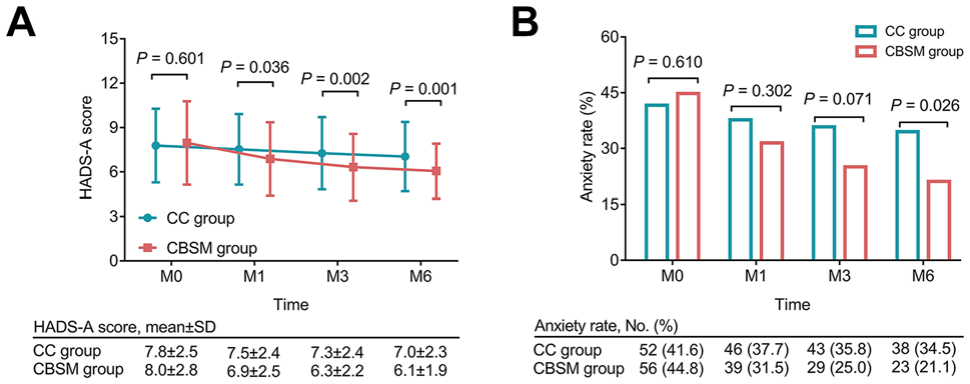
Anxiety in the CBSM group and the CC group. Comparison of HADS-A score at M0, M1, M3, and M6 between the CBSM group and the CC group (**A**); comparison of anxiety rate at M0, M1, M3, and M6 between the CBSM group and the CC group (**B**). The comparisons of HADS-A score and anxiety rate between the two groups were assessed by the student *t*-test and *χ*^2^ test, respectively

The HADS-A score at M0 was not different between the two groups (*P* = 0.601). However, the HADS-A score at M1 (6.9 ± 2.5 vs. 7.5 ± 2.4) (*P* = 0.036), M3 (6.3 ± 2.2 vs. 7.3 ± 2.4) (*P* = 0.002), and M6 (6.1 ± 1.9 vs. 7.0 ± 2.3) (*P* = 0.001) was decreased in the CBSM group vs. the CC group (Fig. 2A). The anxiety rate at M0 (*P* = 0.610) and M1 (*P* = 0.302) did not differ between the two groups, while the anxiety rate at M3 also displayed a decreasing trend in the CBSM group vs. the CC group but did not achieve statistical significance (25.0% vs. 35.8%) (*P* = 0.071). Notably, the anxiety rate at M6 was decreased in the CBSM group vs. the CC group (21.1% vs. 34.5%) (*P* = 0.026) (Fig. 2B).

**Fig. 3:**
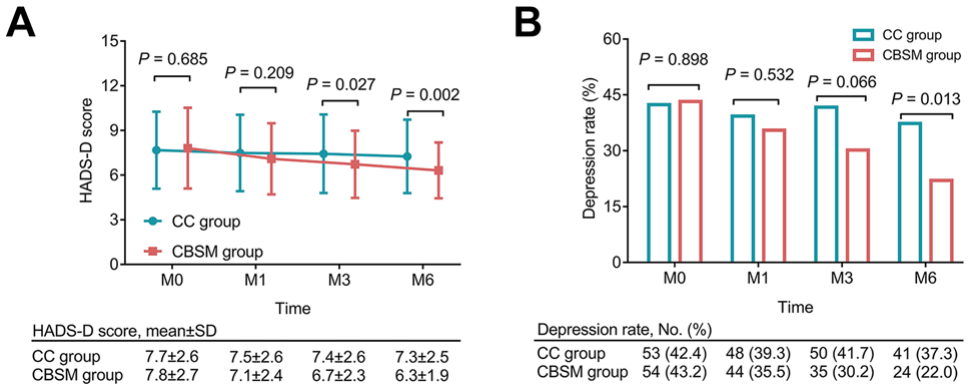
Depression in the CBSM group and the CC group. Comparison of HADS-D score at M0, M1, M3, and M6 between the CBSM group and the CC group (**A**); comparison of depression rate at M0, M1, M3, and M6 between the CBSM group and the CC group (**B**). The comparisons of HADS-D score and depression rate between the two groups were assessed by the student *t*-test and *χ*^2^ test, respectively

The HADS-D score at M0 (*P* = 0.685) and M1 (*P* = 0.209) was not different between the two groups. Nevertheless, the HADS-D score at M3 (6.7 ± 2.3 vs. 7.4 ± 2.6) (*P* = 0.027) and M6 (6.3 ± 1.9 vs. 7.3 ± 2.5) (*P* = 0.002) was reduced in the CBSM group vs. the CC group (Fig. 3A). The depression rate at M0 (*P* = 0.898) and M1 (*P* = 0.532) was not different between the two groups. However, the depression rate at M3 showed a decreasing trend in the CBSM group vs. the CC group but lacked statistical significance (30.2% vs. 41.7%) (*P* = 0.066). Importantly, the depression rate at M6 was declined in the CBSM group vs. the CC group (22.0% vs. 37.3%) (*P* = 0.013) (Fig. 3B).

### Comparison of quality of life between CBSM and CC groups

**Fig. 4:**
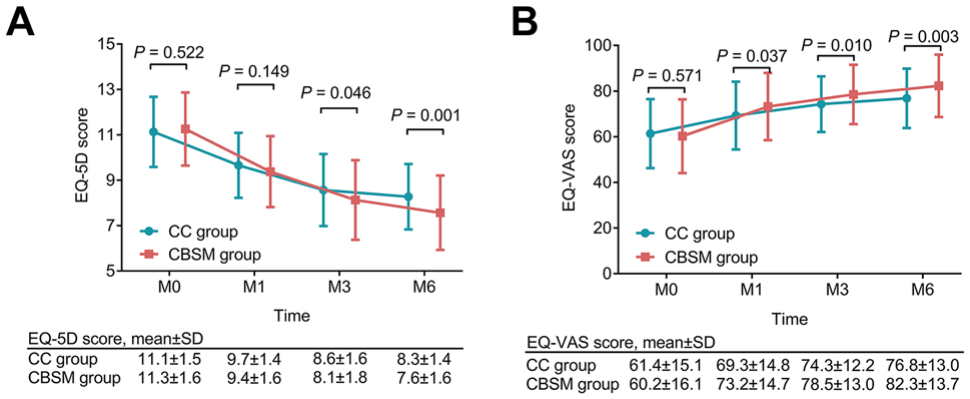
Quality of life in the CBSM group and the CC group. Comparison of EQ-5D score at M0, M1, M3, and M6 between the CBSM group and the CC group (**A**); comparison of EQ-VAS score at M0, M1, M3, and M6 between the CBSM group and the CC group (**B**). The comparisons of EQ-5D score and EQ-VAS score between the two groups were assessed by the student *t*-test

The EQ-5D score at M0 (*P* = 0.522) and M1 (*P* = 0.149) did not differ between the two groups, while the EQ-5D score at M3 (8.1 ± 1.8 vs. 8.6 ± 1.6) (*P* = 0.046) and M6 (7.6 ± 1.6 vs. 8.3 ± 1.4) (*P* = 0.001) was decreased in the CBSM group vs. the CC group (Fig. 4A). The EQ-VAS score at M0 was not different between the two groups (*P* = 0.571). However, the EQ-VAS score at M1 (73.2 ± 14.7 vs. 69.3 ± 14.8) (*P* = 0.037), M3 (78.5 ± 13.0 vs. 74.3 ± 12.2) (*P* = 0.010), and M6 (82.3 ± 13.7 vs. 76.8 ± 13.0) (*P* = 0.003) was raised in the CBSM group vs. the CC group (Fig. 4B).

### Subgroup analysis of anxiety, depression, and quality of life at M6

In patients without anxiety at M0, only the EQ-VAS score at M6 was raised in the CBSM group vs. the CC group (*P* = 0.039). Notably, in patients with anxiety at M0, the HADS-A score, anxiety rate, HADS-D score, depression rate, and EQ-5D score at M6 were decreased but the EQ-VAS score at M6 was elevated in the CBSM group vs. the CC group (all *P* < 0.05) (Table 2).

**Table 2:** Subgroup analyses

Subgroups	CC group	CBSM group	*P* value
**Without anxiety at M0**	*n* = 73	*n* = 69	
HADS-A score at M6, mean ± SD	6.4 ± 2.2	5.9 ± 1.6	0.144
Anxiety rate at M6, No. (%)	11 (16.9)	11 (18.6)	0.802
HADS-D score at M6, mean ± SD	6.9 ± 2.4	6.6 ± 1.9	0.354
Depression rate at M6, No. (%)	22 (33.8)	14 (23.7)	0.215
EQ-5D score at M6, mean ± SD	8.1 ± 1.6	7.6 ± 1.5	0.059
EQ-VAS score at M6, mean ± SD	77.9 ± 13.9	82.9 ± 12.9	0.039
**With anxiety at M0**	*n* = 52	*n* = 56	
HADS-A score at M6, mean ± SD	8.0 ± 2.2	6.3 ± 2.1	< 0.001
Anxiety rate at M6, No. (%)	27 (60.0)	12 (24.0)	< 0.001
HADS-D score at M6, mean ± SD	7.7 ± 2.5	6.0 ± 1.8	< 0.001
Depression rate at M6, No. (%)	19 (42.2)	10 (20.0)	0.019
EQ-5D score at M6, mean ± SD	8.5 ± 1.1	7.6 ± 1.8	0.003
EQ-VAS score at M6, mean ± SD	75.3 ± 11.6	81.6 ± 14.6	0.024
**Without depression at M0**	*n* = 72	*n* = 71	
HADS-A score at M6, mean ± SD	6.7 ± 2.5	6.0 ± 1.9	0.084
Anxiety rate at M6, No. (%)	20 (31.7)	14 (22.2)	0.229
HADS-D score at M6, mean ± SD	6.1 ± 1.9	6.1 ± 1.7	0.921
Depression rate at M6, No. (%)	14 (22.2)	10 (15.9)	0.364
EQ-5D score at M6, mean ± SD	8.0 ± 1.4	7.4 ± 1.5	0.018
EQ-VAS score at M6, mean ± SD	79.4 ± 11.5	83.7 ± 12.9	0.051
**With depression at M0**	n = 53	n = 54	
HADS-A score at M6, mean ± SD	7.6 ± 2.1	6.2 ± 1.8	0.001
Anxiety rate at M6, No. (%)	18 (38.3)	9 (19.6)	0.047
HADS-D score at M6, mean ± SD	8.8 ± 2.3	6.7 ± 2.1	< 0.001
Depression rate at M6, No. (%)	27 (57.4)	14 (30.4)	0.009
EQ-5D score at M6, mean ± SD	8.6 ± 1.5	7.8 ± 1.8	0.018
EQ-VAS score at M6, mean ± SD	73.4 ± 14.2	80.4 ± 14.6	0.021

Comparisons of HADS-A score, HADS-D score, EQ-5D score, and EQ-VAS score between the two groups were assessed by the student *t*-test. The comparisons of anxiety rate and depression rate between the two groups were assessed by the *χ*^2^ test

*CC* control care, *CBSM* cognitive-behavioral stress management, *M0* baseline, *HADS-A* hospital anxiety and depression scale for anxiety, *M6* the 6^th^ months after baseline, *HADS-D* hospital anxiety and depression scale for depression, *SD* standard deviation, *EQ-5D* EuroQol 5D, *EQ-VAS* EuroQol visual analogue scale

In patients without depression at M0, only the EQ-5D score at M6 was declined in the CBSM group vs. the CC group (*P* = 0.018). Importantly, in patients with depression at M0, the HADS-A score, anxiety rate, HADS-D score, depression rate, and EQ-5D score at M6 were reduced, while the EQ-VAS score at M6 was raised in the CBSM group vs. the CC group (all *P* < 0.05) (Table 2).

Regarding marital status, in married patients, HADS-A score (*P* = 0.003), HADS-D score (*P* = 0.002), and EQ-5D score (*P* = 0.001) at M6 were all decreased, but EQ-VAS score (*P* = 0.017) at M6 was increased in the CBSM group compared to the CC group. In single/divorced/widowed patients, HADS-A score, HADS-D score, EQ-5D score, and EQ-VAS score at M6 were not different between the two groups (all *P* > 0.05).

In terms of employment status, in unemployed patients, HADS-A score (*P* = 0.001), HADS-D score (*P* = 0.023), and EQ-5D score (*P* = 0.002) at M6 were all decreased, but EQ-VAS score (*P* = 0.009) at M6 was increased in the CBSM group compared to the CC group. In employed patients, only HADS-D score at M6 was decreased in the CBSM group versus the CC group (*P* = 0.015).

With respect to education level, in patients with an education level of primary school or below, EQ-5D score at M6 (*P* = 0.024) was decreased but EQ-VAS score at M6 (*P* = 0.030) was increased in the CBSM group compared to the CC group. In patients with an education level of middle or high school, HADS-A score at M6 was decreased in the CBSM group compared to the CC group (*P* = 0.005). In patients with an education level of undergraduate or above, HADS-D score (*P* = 0.040) and EQ-5D score (*P* = 0.015) at M6 were deceased but EQ-VAS score (*P* = 0.018) at M6 was increased in the CBSM group versus the CC group (Supplementary Table 1).

### Comparison of accumulating MACE rate between CBSM and CC groups

**Fig. 5:**
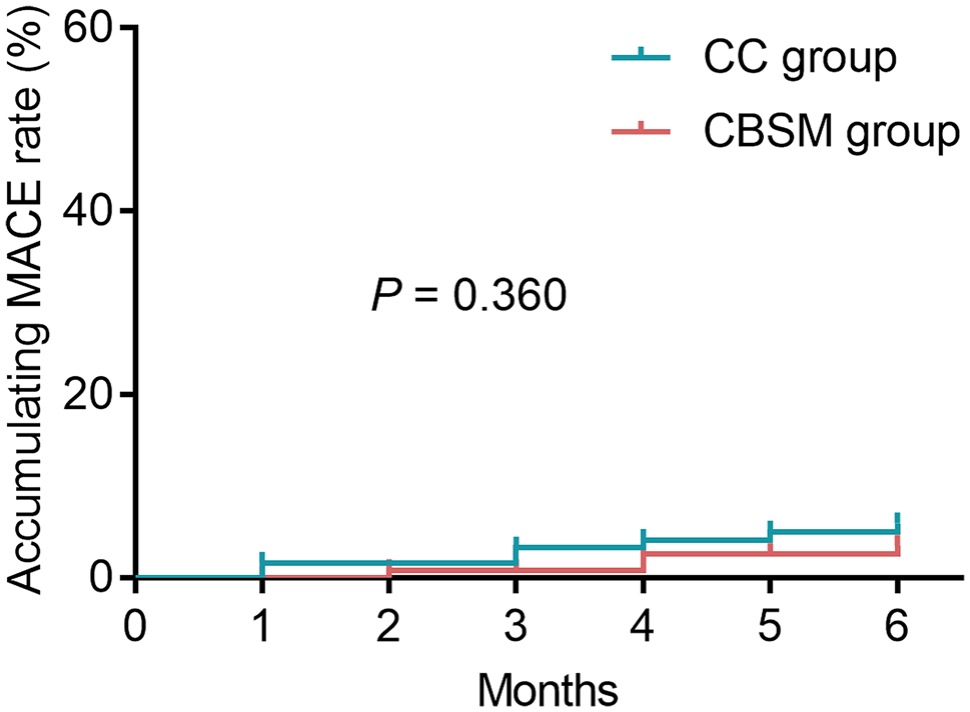
Accumulating MACE in the CBSM group and the CC group. The comparison of accumulating MACE between the two groups was assessed by log-rank test

During the 6-month follow-up, MACE was recorded in the CBSM group and the CC group. It was found that accumulating MACE rate during 6 months was 3.2% in the CBSM group, and it was 5.6% in the CC group. Notably, accumulating MACE rate was not different between the two groups (*P* = 0.360) (Fig. 5).

## Discussion

Anxiety and depression are prevalent in heart disease patients, which may lead to prolonged hospitalization and increased mortality in these patients [11, 28, 29]. The current study found that CBSM intervention reduced anxiety and depression in AMI patients who received PCI compared to CC intervention. The potential reasons would be that (1) CBSM intervention guided and encouraged patients to share their current worries and stresses with others, which was beneficial to relieve anxiety and depression [30, 31]. (2) CBSM intervention helped patients to manage their emotional problems and build their confidence by expressing their troubles with other participants, which was helpful to establish supportive relationships with others and increase their confidence, thereby attenuating anxiety and depression [30]. (3) The relaxation training session of CBSM intervention allowed patients to refresh their minds, eliminate fatigue, and regain strength, which would further result in the reduction of anxiety and depression [32, 33].

Apart from anxiety and depression, reduced quality of life is also a crucial problem in heart disease patients [34]. However, most of the studies mainly focus on the potential of cognitive-behavioral therapy intervention and discover that this intervention improves the quality of life in coronary artery disease patients receiving PCI and heart failure patients [30, 35]. Nevertheless, the potential of CBSM intervention in enhancing the quality of life in AMI patients who undergo PCI needs exploration. The current study discovered that CBSM intervention increased the quality of life in AMI patients who received PCI vs. CC intervention. The reasons behind this might be that (1) anxiety or depression was a crucial dimension to evaluate the quality of life; as discussed above, anxiety and depression were attenuated by CBSM intervention, which might directly or indirectly assist in ameliorating the quality of life in AMI patients who received PCI [30, 32, 33]; (2) CBSM intervention also contained skills-teach session and relaxation training session, which might help to build social relationships with others, increase confidence, relieve stresses, and restore health status, thereby enhancing the quality of life [36]. Taken together, quality of life could be improved by CBSM intervention in AMI patients who underwent PCI.

Interestingly, the subgroup analysis found that patients with anxiety and depression at baseline might be benefited more from CBSM intervention in attenuating anxiety and depression, along with enhancing the quality of life at M6. The potential arguments might be that patients with anxiety and depression at baseline had severe psychological conditions; thus, the benefit of CBSM intervention might be stronger; in addition, patients could learn about this intervention after months; thus, they could conduct this intervention in their daily life, resulting in the reduction of anxiety and depression, and the enhancement of the quality of life at M6 [37, 38]. Notably, the current study also discovered that accumulating MACE was not affected by CBSM in AMI patients who underwent PCI. The speculation would be that limited by the follow-up duration, the occurrence rates of MACE were low, and the benefit of CBSM intervention might need a longer time to realize. Taken together, the benefit of CBSM intervention in MACE was not obvious in AMI patients who received PCI.

Although several interesting findings had been revealed, the limitations could not be omitted. Firstly, the long-term benefit of CBSM intervention in reducing anxiety and depression, along with ameliorating the quality of life in AMI patients who underwent PCI should be further explored. Secondly, the HADS score, EQ-5D score, and EQ-VAS score were self-assessed, which might lead to assessment bias. Thirdly, some individual-based psychotherapeutic interventions had been reported to improve mental health and quality of life [39–41]; however, in this study, the CBSM intervention was carried out in a group setting, and the impact of the individual-based CBSM intervention on enhancing mental condition and the quality of life in AMI patients who received PCI was unknown and could be further explored. Fourthly, the follow-up duration was short; thus, the potential of CBSM intervention on reducing MACE in AMI patients who underwent PCI should be explored by further studies with a longer follow-up duration.

In conclusion, CBSM is a potential intervention in ameliorating anxiety, depression, and quality of life but does not affect MACE in AMI patients who undergo PCI. More shreds of evidence are required to validate the findings of this study.

### Supplementary Information

Below is the link to the electronic supplementary material.

Supplementary file1 (DOCX 21 KB)
