# Supplementary material for: Implication of cognitive-behavioral stress management on anxiety, depression, and quality of life in acute myocardial infarction patients after percutaneous coronary intervention: a multicenter, randomized, controlled study
Source: Ir J Med Sci. 2023 Jun 23;193(1):101–9. doi: 10.1007/s11845-023-03422-6 (PMC10808172; doi:10.1007/s11845-023-03422-6)
Supplement: Supplementary file 1 — Supplementary file1 (DOCX 21 KB) [file 11845_2023_3422_MOESM1_ESM.docx]

**Supplementary Table 1.** Subgroup analyses based on marital status, employment status, and education level.

| Subgroups | CC group | CBSM group | *P* value |
| --- | --- | --- | --- |
| **Married** | n = 95 | n = 90 |  |
| HADS-A score at M6, mean±SD | 6.9±2.2 | 6.0±1.9 | 0.003 |
| HADS-D score at M6, mean±SD | 7.4±2.4 | 6.2±2.0 | 0.002 |
| EQ-5D score at M6, mean±SD | 8.4±1.4 | 7.5±1.6 | 0.001 |
| EQ-VAS score at M6, mean±SD | 75.5±13.2 | 80.8±14.3 | 0.017 |
| **Single/divorced/widowed** | n = 30 | n = 35 |  |
| HADS-A score at M6, mean±SD | 7.4±2.8 | 6.3±1.8 | 0.073 |
| HADS-D score at M6, mean±SD | 7.0±2.6 | 6.6±1.4 | 0.439 |
| EQ-5D score at M6, mean±SD | 8.0±1.4 | 7.7±1.6 | 0.479 |
| EQ-VAS score at M6, mean±SD | 80.7±11.7 | 86.1±11.2 | 0.079 |
| **Employed** | n = 32 | n = 31 |  |
| HADS-A score at M6, mean±SD | 6.6±2.1 | 6.0±1.8 | 0.221 |
| HADS-D score at M6, mean±SD | 7.4±2.4 | 6.0±1.4 | 0.015 |
| EQ-5D score at M6, mean±SD | 8.2±1.7 | 7.6±1.7 | 0.206 |
| EQ-VAS score at M6, mean±SD | 77.2±14.4 | 82.7±12.8 | 0.145 |
| **Unemployed** | n = 93 | n = 94 |  |
| HADS-A score at M6, mean±SD | 7.2±2.4 | 6.1±1.9 | 0.001 |
| HADS-D score at M6, mean±SD | 7.2±2.5 | 6.4±2.0 | 0.023 |
| EQ-5D score at M6, mean±SD | 8.3±1.4 | 7.6±1.6 | 0.002 |
| EQ-VAS score at M6, mean±SD | 76.7±12.6 | 82.2±14.0 | 0.009 |
| **Primary school or below** | n = 30 | n = 24 |  |
| HADS-A score at M6, mean±SD | 7.0±1.6 | 6.4±1.6 | 0.238 |
| HADS-D score at M6, mean±SD | 7.5±2.3 | 6.7±2.1 | 0.238 |
| EQ-5D score at M6, mean±SD | 8.5±1.3 | 7.5±1.5 | 0.024 |
| EQ-VAS score at M6, mean±SD | 75.0±15.6 | 84.2±10.2 | 0.030 |
| **Middle or high school** | n = 60 | n = 69 |  |
| HADS-A score at M6, mean±SD | 7.3±2.5 | 6.1±2.1 | 0.005 |
| HADS-D score at M6, mean±SD | 7.0±2.4 | 6.2±1.9 | 0.061 |
| EQ-5D score at M6, mean±SD | 8.2±1.5 | 7.7±1.7 | 0.144 |
| EQ-VAS score at M6, mean±SD | 80.4±10.8 | 82.5±14.7 | 0.402 |
| **Undergraduate or above** | n = 35 | n = 32 |  |
| HADS-A score at M6, mean±SD | 6.7±2.5 | 5.8±1.5 | 0.118 |
| HADS-D score at M6, mean±SD | 7.5±2.7 | 6.2±1.7 | 0.040 |
| EQ-5D score at M6, mean±SD | 8.3±1.5 | 7.3±1.6 | 0.015 |
| EQ-VAS score at M6, mean±SD | 72.5±12.7 | 80.7±13.6 | 0.018 |

CC, control care; CBSM, cognitive behavioral stress management; HADS-A, hospital anxiety and depression scale for anxiety; M6, the 6^th^ months after baseline; HADS-D, hospital anxiety and depression scale for depression; SD, standard deviation; EQ-5D, EuroQol 5D; EQ-VAS, EuroQol visual analogue scale.

Footnote: comparisons of HADS-A score, HADS-D score, EQ-5D score, and EQ-VAS score between the two groups were assessed by the student *t*-test.
